# Supplementary material for: Mixed responses to targeted therapy driven by chromosomal instability through p53 dysfunction and genome doubling
Source: Nat Commun. 2024 Jun 13;15:4871. doi: 10.1038/s41467-024-47606-9 (PMC11176322; doi:10.1038/s41467-024-47606-9)
Supplement: Supplementary file 1 — Supplementary Information [file 41467_2024_47606_MOESM1_ESM.pdf]

Supplementary information for

Mixed responses to targeted therapy driven by chromosomal instability through p53 dysfunction and genome doubling

Sebastijan Hobor[1*], Maise Al Bakir[1*], Crispin T. Hiley[1,2,3*], Marcin Skrzypski[1,2,3,4*], Alexander M. Frankell[1,2], Bjorn Bakker[1,5], Thomas B. K. Watkins[1], Aleksandra Markovets[6], Jonathan R Dry[7], Andrew P. Brown[7], Jasper van der Aart[8], Hilda van den Bos[5], Diana Spierings[5], Dahmane Oukrif[9], Marco Novelli[9], Turja Chakrabarti[10], Adam H. Rabinowitz[11], Laila Ait Hassou[12], Saskia Litière[13], D. Lucas Kerr[10], Lisa Tan[10], Gavin Kelly[13], David A Moore[2,14], Matthew J Renshaw[15], Subramanian Venkatesan[1], William Hill[1], Ariana Huebner[1,2,16], Carlos Martínez-Ruiz[2,16], James R. M. Black[2,16], Wei Wu[10,] Mihaela Angelova[1], Nicholas McGranahan[2,16], Julian Downward[17], Juliann Chmielecki[7], Carl Barrett[7], Kevin Litchfield[1], Su Kit Chew[1,2], Collin M. Blakely[10], Elza C. de Bruin[8], Floris Foijer[5], Karen H. Vousden[18], Trever G. Bivona[10,19], TRACERx consortium[‡], Robert E. Hynds[1,2], Nnennaya Kanu[✉2#], Simone Zaccaria[✉2,20#], Eva Grönroos[✉1#] and Charles Swanton[✉1,2,3#]

1. Cancer Evolution and Genome Instability Laboratory, The Francis Crick Institute, 1 Midland Rd, London NW1 1AT, UK
2. Cancer Research UK Lung Cancer Centre of Excellence, University College London Cancer Institute, Paul O'Gorman Building, 72 Huntley Street, London, WC1E 6BT, UK
3. Department of Medical Oncology, University College London Hospitals, 235 Euston Rd, Fitzrovia, London, United Kingdom, NW1 2BU, UK

4. Department of Oncology and Radiotherapy, Medical University of Gdańsk, ul. Mariana Smoluchowskiego 17, 80-214, Gdańsk, Poland
5. European Research Institute for the Biology of Ageing, University of Groningen, University Medical Center Groningen, A. Deusinglaan 1, Groningen 9713, the Netherlands.
6. Oncology Data Science, Oncology R&D, AstraZeneca, Boston, MA, USA
7. Late Development, Oncology R&D, AstraZeneca, Boston, MA, USA
8. Research and Early Development, Oncology R&D, AstraZeneca, Cambridge, UK
9. Research Department of Pathology, University College London Medical School, University Street, London WC1E 6JJ, UK
10. Department of Medicine, University of California, San Francisco, CA 94158, USA
11. Furlong Laboratory, EMBL Meyerhofstraße 1, 69117 Heidelberg, Germany
12. European Organization for Research and Treatment of Cancer, Brussels, Belgium
13. Bioinformatics & Biostatistics; Francis Crick Institute, London, UK
14. Department of Cellular Pathology, University College London Hospitals, London, UK
15. Advanced Light Microscopy, The Francis Crick Institute, 1 Midland Rd, London NW1 1AT, UK
16. Cancer Genome Evolution Research Group, Cancer Research UK Lung Cancer Centre of Excellence, University College London Cancer Institute, London, UK
17. Oncogene Biology Laboratory, The Francis Crick Institute, 1 Midland Rd, London NW1 1AT, UK
18. p53 and Metabolism Laboratory, The Francis Crick Institute, 1 Midland Rd, London NW1 1AT, UK
19. Chan-Zuckerberg Biohub
20. Computational Cancer Genomics Research Group, University College London Cancer Institute, London, UK

‡ A full list of consortium members appears at the end of the paper.

* These authors contributed equally to this work.

# These authors jointly supervised this work.

✉ n.kanu@ucl.ac.uk

✉ s.zaccaria@ucl.ac.uk

✉ Eva.Gronroos@crick.ac.uk

✉ Charles.Swanton@crick.ac.uk

This File includes:
Supplementary Figures 1-10
Supplementary References
Supplementary Tables 1-3

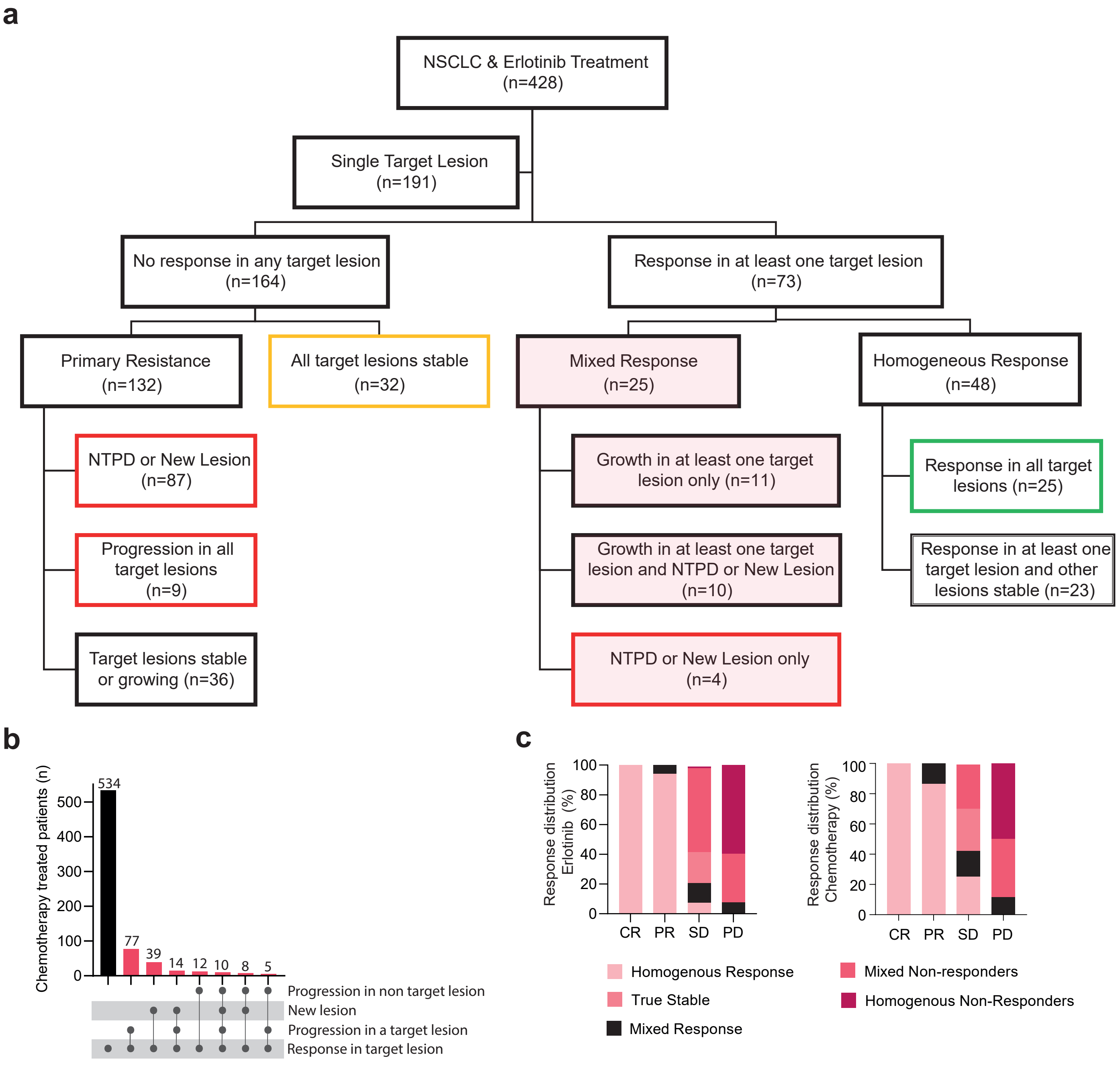

Supplementary Fig. 1: Heterogenous patient responses are observed across multiple RECIST categories in NSCLC. a, Consort diagram of responses in erlotinib treated NSCLC patients in the RECIST database on a per lesion basis. Response and progression defined as per Reiter et al[1]. Tumor responses defined as PD by RECISTv1.1 outlined in red border, PR/CR outlined in green border and SD in orange. Tumor responses outlined in black border contained RECISTv1.1 responses that include PR/SD/PD dependent on the sum of diameters of all target lesions. **b**, Heterogeneous Responses in the RECIST database were analysed using response criteria defined by Reiter et al[1]. Patients with at least two lesions where one shrank by at least 30% were included in the analysis. The number of patients with homogeneous responses are shown in black for patients receiving chemotherapy. The different patterns of progression seen in patients with a heterogeneous response are show in pink. **c**, Bar chart showing the proportion of heterogenous responses found in patients classified according to the RECISTv1.1 criteria treated with erlotinib (left panel) or chemotherapy (right panel). Homogenous responses and heterogenous responses are defined in the main manuscript. True stable; no lesions reducing in size by ≥ 30% and no lesions increasing in size by ≥ 10% with no new lesions and no evidence of non-target lesion progression. Heterogenous non-responders; at least one lesion (but not all lesions) increasing in size by ≥ 10% and no lesions reducing in size by ≥ 30% with or without the presence of new lesions or evidence of non-target lesion progression. Homogenous non-responders; all lesions increasing in size by ≥ 10% with or without the presence of new lesions or evidence of non-target lesion progression. Source data are provided as a Source Data file.

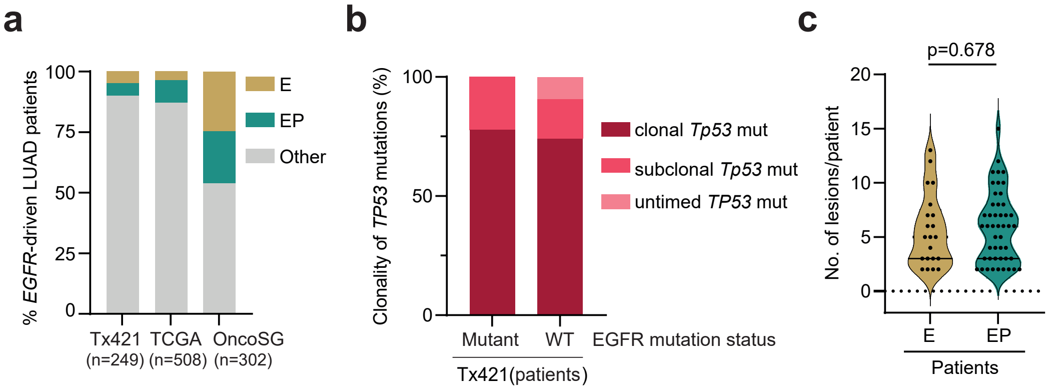

d

EGFR Mutation			
	E (N=35) patients	EP (N=82) patients	p value
**Brain metastasis**			
Absent/Present	26 (74.3%)/9 (25.7%)	58 (70.7%)/24 (29.3%)	0.696
**Visceral metastasis**			
Absent/Present	13 (37.1%)/22 (62.9%)	30 (36.6%)/52 (63.4%)	0.954
**Liver metastasis**			
Absent/Present	27 (77.1%)/8 (22.9%)	59 (72.0%)/23 (28.0%)	0.56
**Smoking status**			
FORMER/NEVER	9 (25.7%)/26 (74.3%)	26 (31.7%)/56 (68.3%)	0.517
**WHO Performance Status**			
0/1	10 (28.6%)/25 (71.4%)	28 (34.1%)/54 (65.9%)	0.555
**Sum of longest diameters (mm)**			
Mean (SD)	58.257 (36.066)	54.195 (36.432)	0.581
Range	15.000 - 156.000	11.000 - 229.000	

*** investigator assessed sum of longest diameter of target lesions at baseline scan

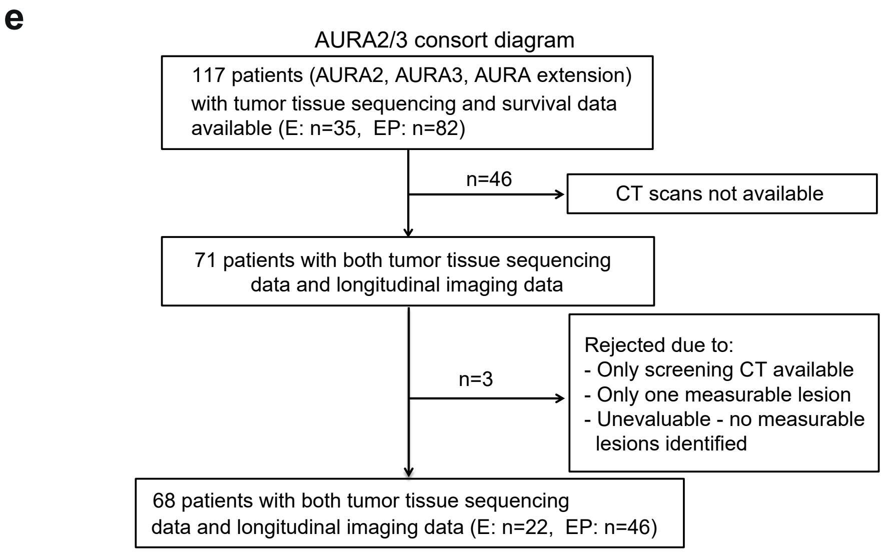

Supplementary Fig. 2: Clinical characteristics of patients participating in observational and interventional trial cohorts. **a**, Bar charts showing the proportion of LUAD patients with mutated EGFR (E) and EGFR with concomitant TP53 pathway disruption (EP) in predominantly western (Tx421, TCGA) and eastern (OncoSG) cohorts. **b**, Graph depicting the clonality of TP53 mutations for the 249 LUAD patients within Tx421. Of the 25 LUAD Tx421 patients with clonal EGFR mutations, 7 had clonal TP53 mutations and 2 had a subclonal TP53 mutation. Of the 224 LUAD patients with wildtype EGFR status, 86 had clonal TP53 mutations, 19 subclonal TP53 mutations and 11 TP53 mutations were untimed. The remaining 108 patients were TP53 wild type. **c**, Violin plots showing no significant difference in the number of lesions per patient from 22 patients with E tumors (yellow) and 46 patients with EP tumors (green) respectively. Each dot represents one patient (p=0.678, two-sided Mann-Whitney *U* test). **d**, Table showing non-significant differences in patient clinical parameters (two way ANOVA and two sided chi squared test for continuous and categorical data respectively). **e**, Consort diagram showing the selection of AURA2 and AURA3 patients. Source data are provided as a Source Data file.

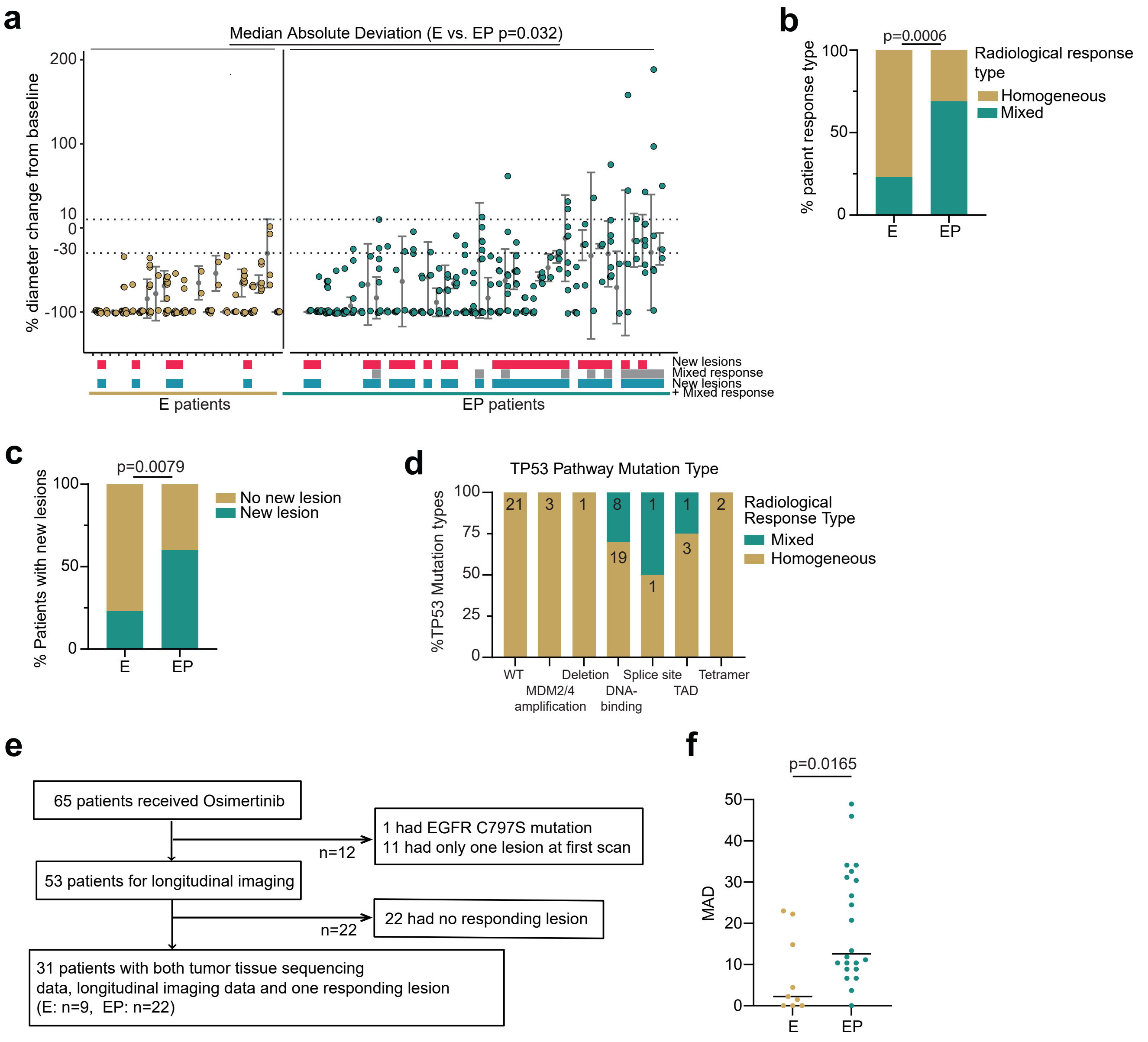

Supplementary Fig. 3: Mixed responses to Osimertinib are more common in patients with TP53 pathway disruption. a, Individual best tumor response within six months on osimertinib treatment, presented as % change in CT measured tumor length. Each x axis tick represents one patient (E n=22, EP n=42). Dotted lines show the Reiter et al.[1] criteria for response (-30%) and progression (10%) respectively. Grey dots and whiskers represent median change in tumor size and variability around the median value using the median absolute deviation (MAD) for each patient. Boxes under-neath the graph indicate the occurrences of new lesions (red box), heterogeneous responses of exist-ing lesions, as defined by Reiter et al1 (grey box), and patients with mixed responses with or without the occurrences of new lesions (blue box). **b,** Bar chart of the proportion of homogenous (yellow) and mixed (green) responses to osimertinib in patients with E (n=22) or EP (n=42) tumors at the time point of best response (p=0.0006, two-sided Fisher's exact test). **c,** Bar chart of the proportion of E (n=22) and EP (n=42) patients with new lesions during osimertinib treatment at the time point of best response (p=0.0079 two-sided Fisher's exact test). **d,** Bar chart of the proportion of homogenous (yellow) and heterogenous (green) responses to osimertinib in patients with different TP53 mutations. The num-bers in the bars denote the number of patients in the different groups. **e,** Consort diagram showing the selection of UCSF patients. **f,** Dot plot showing the Median absolute deviation of the percentage diameter change from baseline of tumor responses within each patient in the E (n=9) and EP (n=22) group (p=0.0165, two-sided Wilcoxon test). Source data are provided as a Source Data file.

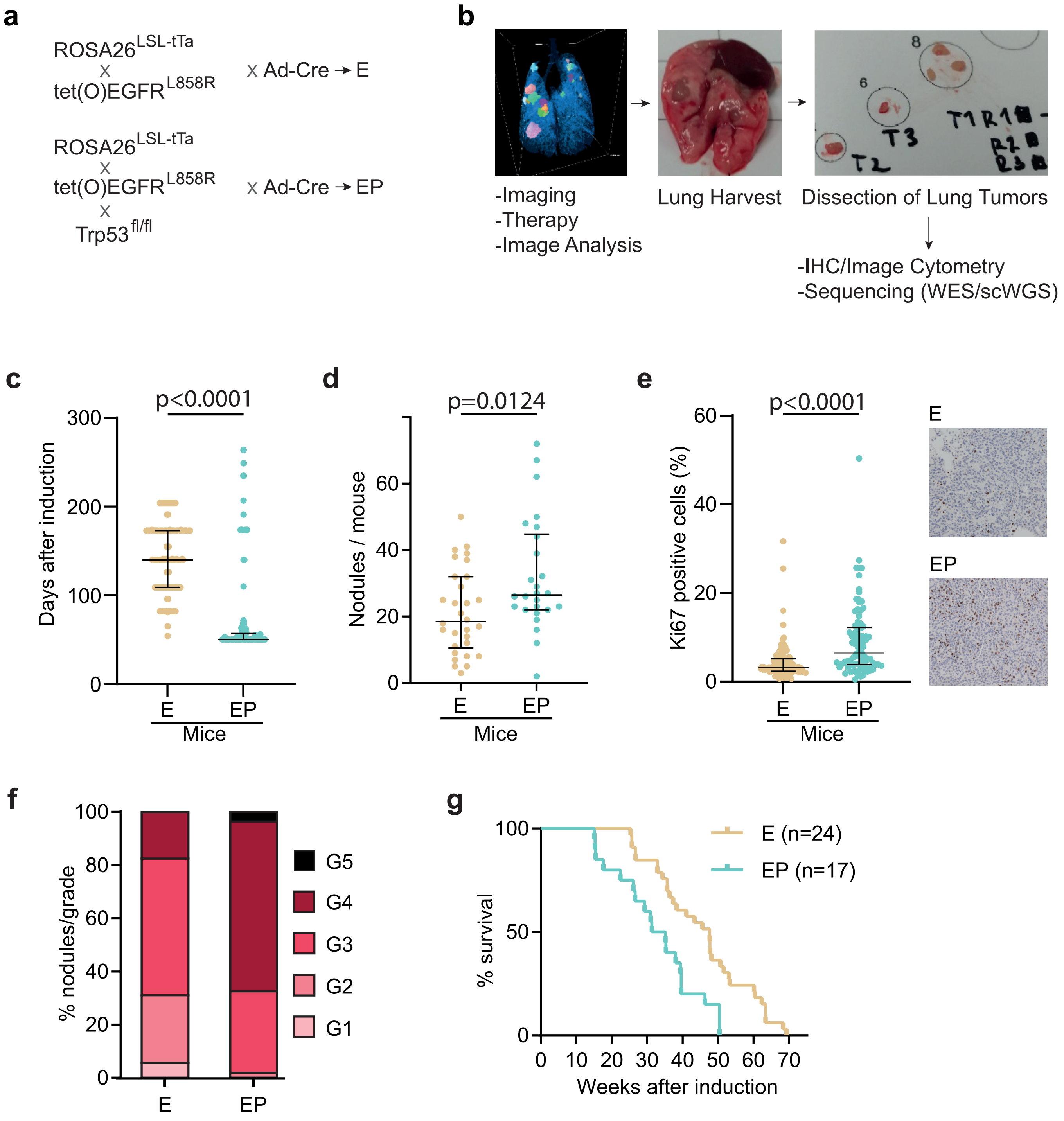

Supplementary Fig. 4: Trp53 disruption results in a more aggressive phenotype in murine models of NSCLC. a, Schematic of the GEMMs crosses for *in vivo* experiments. **b**, Schematic of *in vivo* experimental workflow, Immunohistochemistry (IHC), Single Cell whole genome sequencing (scWGS). **c**, Dot plot showing the time to tumor detection after recombination in E (n=30) and EP (n=26) mice (p<0.0001, two-sided Mann-Whitney *U* test. Data are presented as the median with the interquartile range). **d**, Dot plot showing number of identifiable nodules per mouse in micro-CT scans in E (n=30) and EP (n=26) mice (p=0.0124 two-sided Mann-Whitney *U* test. Data are presented as the median with the interquartile range). **e**, Dot plot showing the percentages of Ki67 positive cells in E (n=116) and EP (n=85) mouse tumors, (p<0.0001 two-sided Mann-Whitney *U* test. Data are presented as the median with the interquartile range). **f**, Bar chart showing the percent of nodules per histological grade (G1=Well differentiated, G2=Well to moderately differentiated, G3=Moderately differentiated, G4=Moderately to poorly differenti-ated, G5=Poorly differentiated) for E (n=3 mice, 142 tumors) and EP (n=5 mice, 166 tumors) mice, (p=0.1704, two-sided Kruskal-Wallis test). **g**, Kaplan-Meier survival analysis demonstrating the difference in overall survival between E (n=33, yellow line) and EP (n=20, green line) mice. (p=0.0014, HR 2.55, 95%CI: 1.235-4.744, log-rank Mantel-Cox test). Source data are provided as a Source Data file.

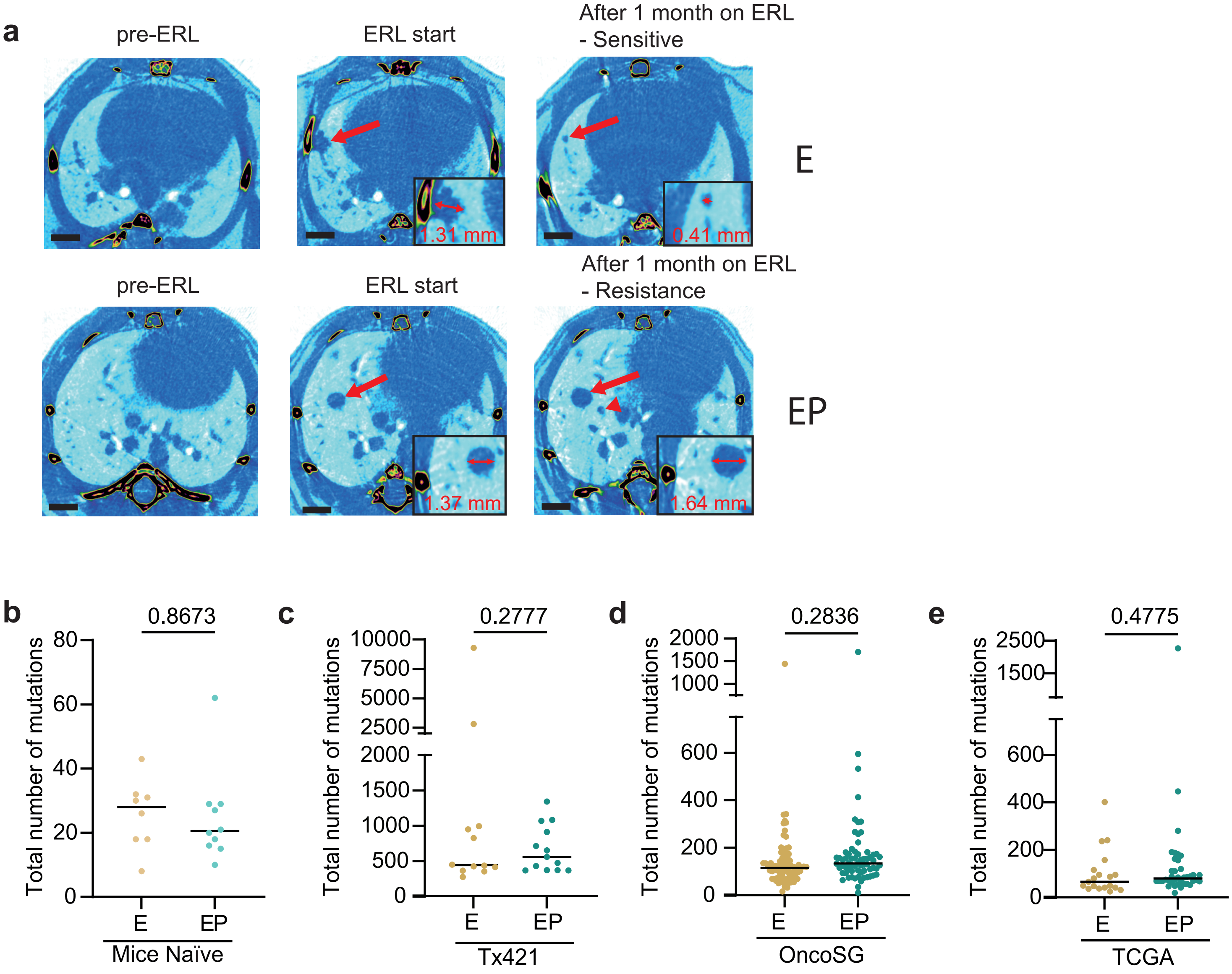

Supplementary Fig. 5: Trp53/TP53 pathway disruption does not affect total mutation burden in murine or human NSCLC tumours. **a**, Representative micro-CT scans from an E (top row) and EP (bottom row) mouse during erlotinib (ERL) treatment. Left images: initial scans. Middle images; scan at the start of ERL therapy. Right images; scan after one month of ERL therapy. Scale bar: 2mm. Red arrows show tracked tumors, arrowhead shows a resistant tumor undetectable at start of treatment. **b**, Dot plot showing a non-significant difference in mutation burden between E (n=9, yellow) and EP (n=10, turquoise) erlotinib resistant mouse tumors (p=0.8673, two-sided t-test, the line indicates the median). **c**, Dot plot showing a non-significant difference in mutation burden between patients with E (n=12, yellow) or EP (n=13, turquoise) tumors in the TRACERx cohort (p=0.2777, two-sided t-test). **d**, Dot plot showing a non-significant difference in mutation burden between patients with E (n=75, yellow) or EP (n=66, turquoise) tumors in the OncoSG cohort (p=0.2836, two-sided t-test, the line indicates the median). **e**, Dot plot showing a non-significant difference in mutation burden between patients with E (n=19, yellow) or EP (n=38, turquoise) tumors in the TCGA cohort (p=0.4775, two-sided t-test, the line indicates the median). Source data are provided as a Source Data file.

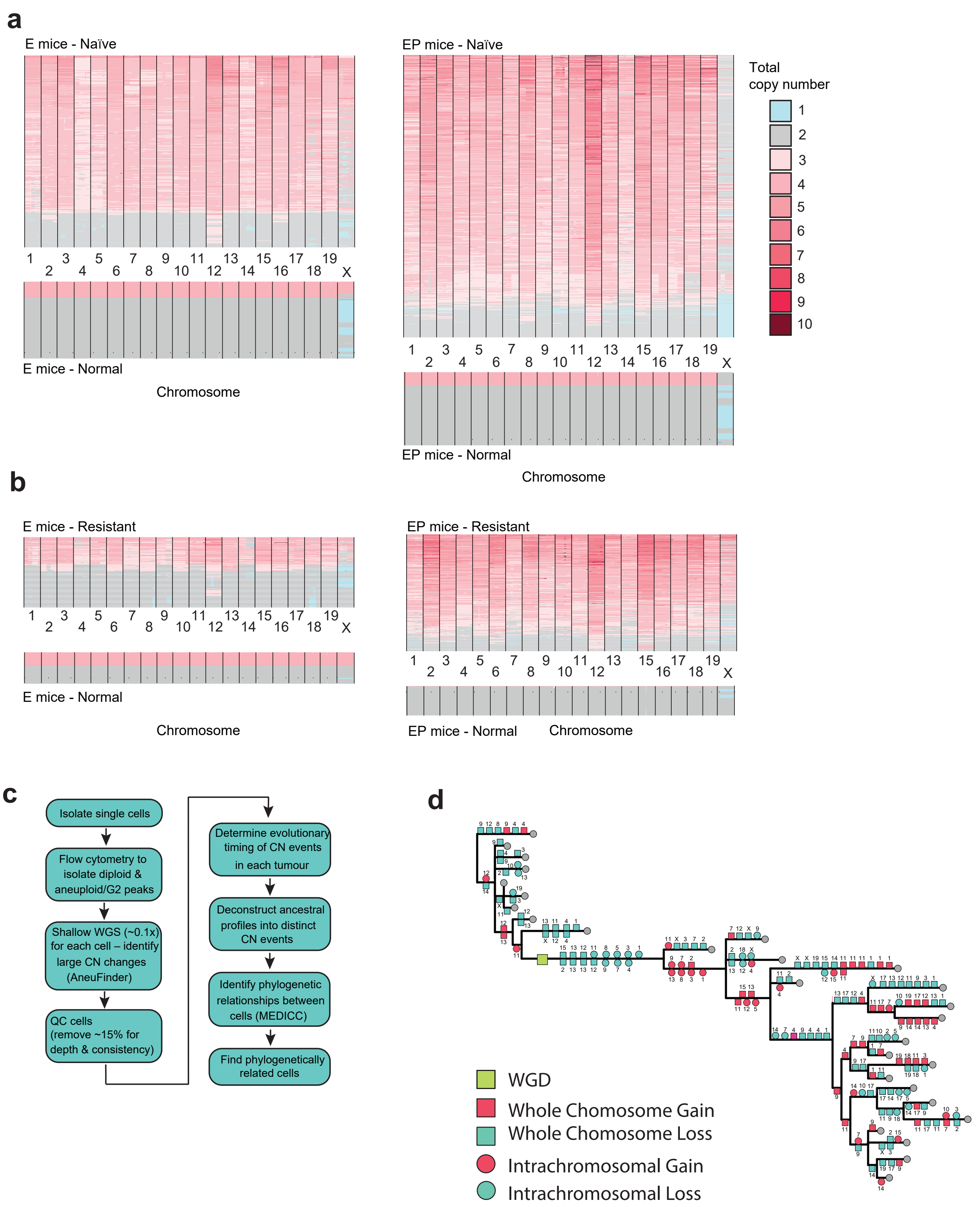

Supplementary Fig. 6: Increased cell to cell variation with Trp53 disruption but clonal expansion is facilitated by whole genome doubling. a, Total copy numbers (colours) are inferred across the whole genome (columns) from scWGS data derived from 338 and 455 single cells (rows) from eight E (left) and seven EP (right) naïve tumors. **b**, Total copy numbers (colours) are inferred across the whole genome (columns) from scWGS data derived from 175 and 231 single cells (rows) from seven E (top) and ten EP (bottom) resistant tumors. **c**, Flow chart showing single cell analysis. **d**, A phylogenetic tree where each terminal node (leaf, grey circle) represents a single cell from a murine lung tumour and genetic 'relatedness' is inferred using shared somatic copy number alterations (SCNAs) determined using single cell shallow WGS and MEDICC (see methods). Internal nodes of the tree are inferred by MEDICC. The length of each edge on the tree represents the number of SCNA events that have occurred during the transition from the parental to the daughter node in the phylogeny. These events are also represented by coloured squares and circles to the left of each edge with the effected chromosome denoted. Source data are provided as a Source Data file.

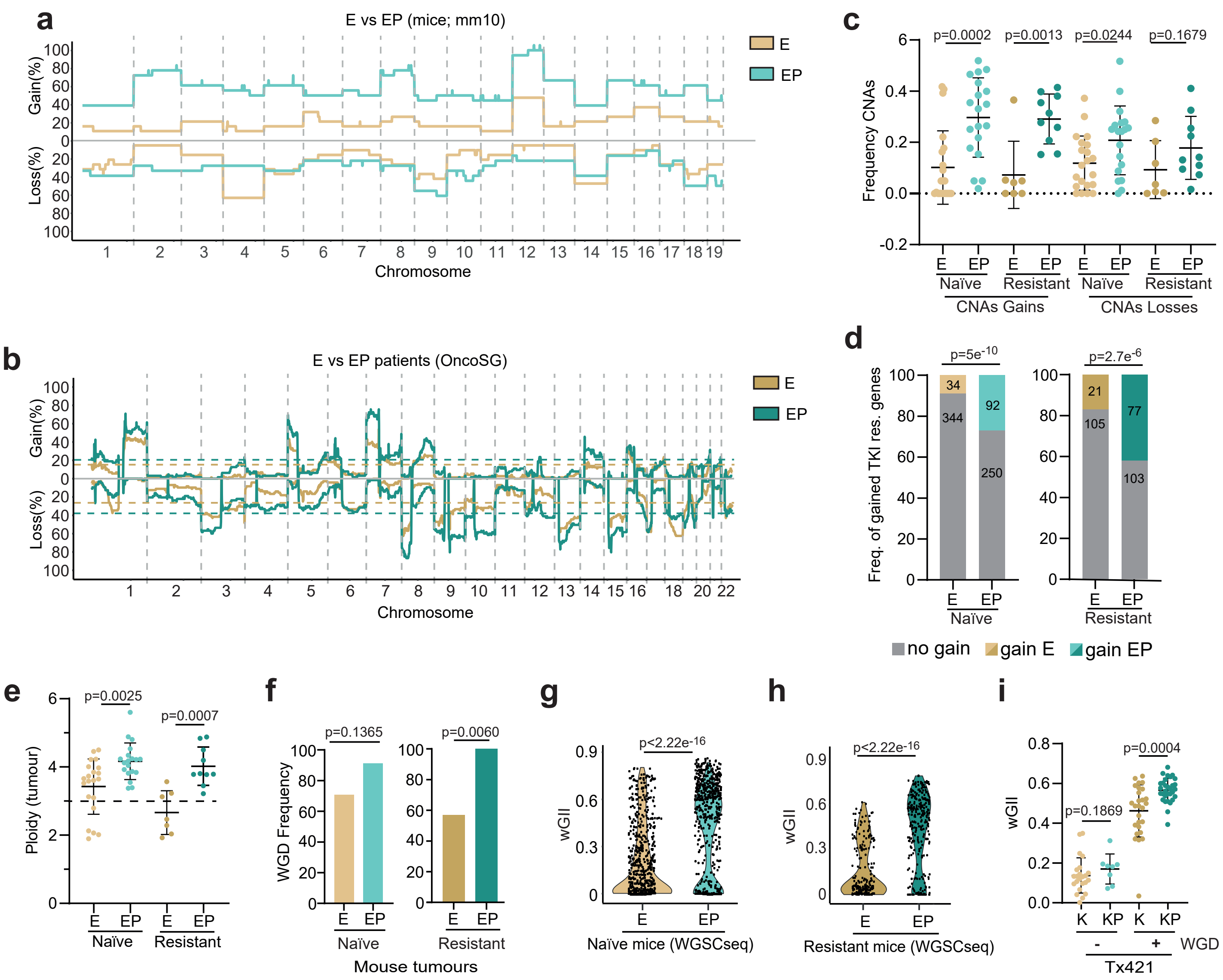

Supplementary Fig. 7: Trp53/TP53 disruption is associated with increased copy number aberration frequency, whole genome doubling and increased genome instability. a, The proportion of the genome affected by SCNAs in mouse E (yellow n=21) and EP (green n=19) tumors respectively. **b**, The proportion of the genome affected by SCNAs in human E (yellow n=75) and EP (turquoise n=66) tumors respectively. **c**, Frequency of SCNAs gains and losses inferred (defined relative to cell ploidy) from naïve and resistant E (yellow, n=21 naïve, n=7 resistant) and EP (green n=19 naïve, n=10 resistant) tumors (naïve E vs EP gains p=0.0002; resistant E vs EP gains p=0.0013; naïve E vs EP losses p=0.0244; resistant E vs EP losses p=0.1679, two-sided t test. Data are presented as mean values +/- SD). **d**, Bar chart showing the frequency of gains in genes known to be involved in TKI resistance, in both treatment-naïve ($p=5e^{-10}$, two-sided Chi-squared test) and resistant E and EP mouse tumors ($p=2.7e^{-6}$; E, n=21 naïve, n=7 resistant and EP n=19 naïve, n=10 resistant two-sided Chi squared test) **e**, Average cell ploidy for every tumor across the E and EP mouse cohorts in both treatment-naïve (E, n=21 and EP n=19, p=0.0025, two sided Mann-Whitney *U* test) and resistant (E, n=7 and EP n=10, p=0.0007, two sided Mann-Whitney *U* test;) cohorts. Data are presented as mean values +/- SD. Based on published pan-cancer studies[2], the values of ploidy likely consistent with the absence vs. occurrence of WGD are separated (dashed black line). **f**, Bar charts showing WGD frequencies in lesions from both treatment-naïve (p=0.1365, two-sided Chi-squared test) and resistant (p=0.0060 two-sided Chi-squared test). E and EP mouse tumors (E, n=21 naïve, n=7 resistant and EP n=19 naïve, n=10 resistant). Violin plot showing weighted Genome Instability Index (wGII) in **g**, naïve E (n=21) and EP (n=19) mouse tumors (p<2.22e-16 two sided Mann-Whitney U test) and **h**, resistant E (n=7) and EP (n=10) mouse tumors (p<2.22e-16 two-sided Mann-Whitney U test). **i**, wGII in nonWGD KRAS (K, n=23) and KRAS/p53 pathway (KP, n=8) mutant tumours (p=0.1869, two-sided Mann-Whitney *U* test) and WGD tumors from KRAS (n=27) and KRAS/p53 (n=31) pathway mutant tumours in the Tx421 cohort (p=0.0004, two-sided Mann-Whitney *U* test. Data are presented as mean values +/- SD). Source data are provided as a Source Data file.

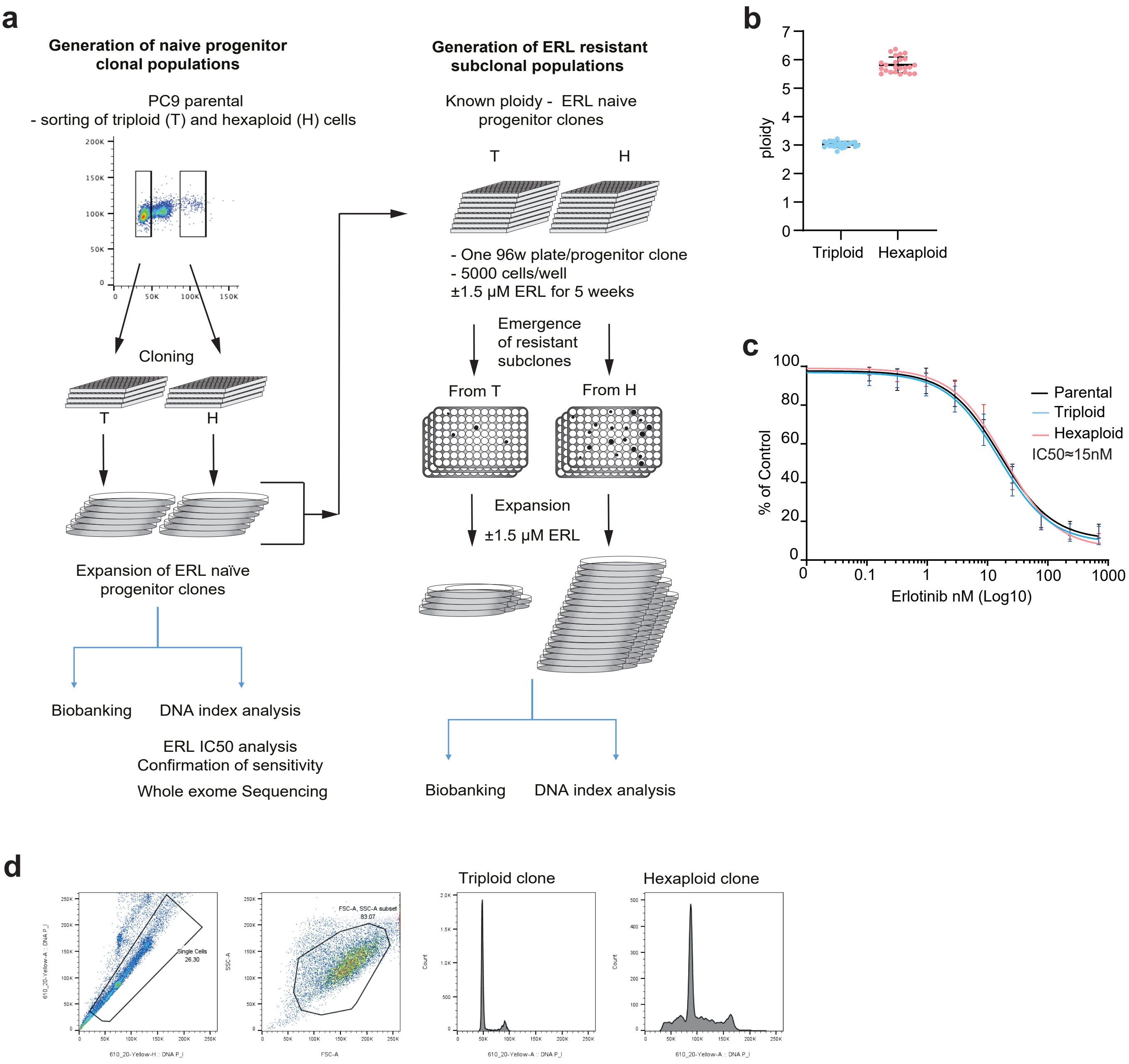

Supplementary Fig. 8: Isolating and characterizing triploid and hexaploid PC9 cells.
a, Schematic overview of workflow for PC9 clone derivation (left panel) and resistant PC9 sub-clone derivation (right panel). The flow cytometry gating strategy to sort triploid and hexaploid clones is indicated in the top left panel. **b**, Inferred ploidy from flow cytometry analysis of DNA content from single cell cloned triploid (T, n=24, blue) and hexaploid (H, n=24, red) propagated PC9 clones. **c**, Dose-response curves of PC9 parental (black line), Triploid (T, n=24, blue line) and Hexaploid (H, n=24 red line) clones. Cells were cultured in increasing concentrations of erlotinib for 96 hours, after which cell viability was measured using CellTitre-Blue. Each data point represents the mean ±SEM of 3 biologically independent experiments with 3 technical replicates per data point. **d**, Gating strategy to infer cell ploidy.

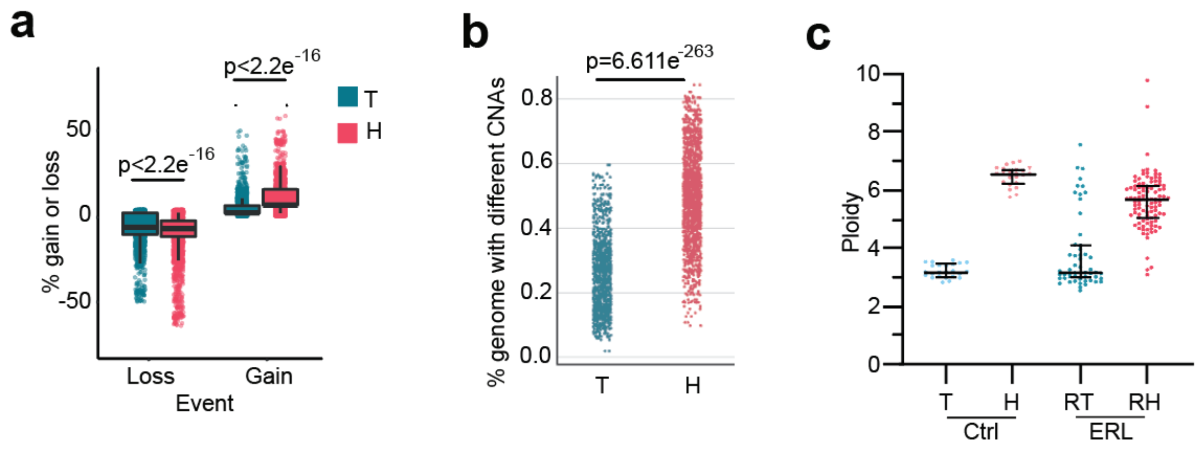

Supplementary Fig.9: Increased prevalence of copy number aberrations in whole genome doubled PC9 clones resistant to EGFR tyrosine kinase inhibition. a, Percentage of gains and losses identified in 34 triploid- and 40 hexaploid-derived resistant PC9 subclones, (two-sided Wilcoxon test, p<2.2e-16) **b**, The fraction of the genome with different SCNAs (y-axis) is measured between every pair of either triploid (blue and hexaploid (red) resistant daughter clones (x-axis), p= 6.611e-263 two-sided Mann-Whitney U test. **c**, Inferred ploidy from flow cytometry analysis of DNA content from triploid (T, n=24, blue), hexaploid (H, n=24, red) progenitor clones and resistant daughter clones derived from triploid (RT, n=53, dark blue) and hexaploid (RH, n=101, dark red) PC9 clones after five weeks in culture with or without erlotinib. Each data point represents one clonal population. The median and interquartile range is indicated in the figure. Source data for this figure are provided as a Source Data file and at doi.org/10.5281/zenodo.10156620.

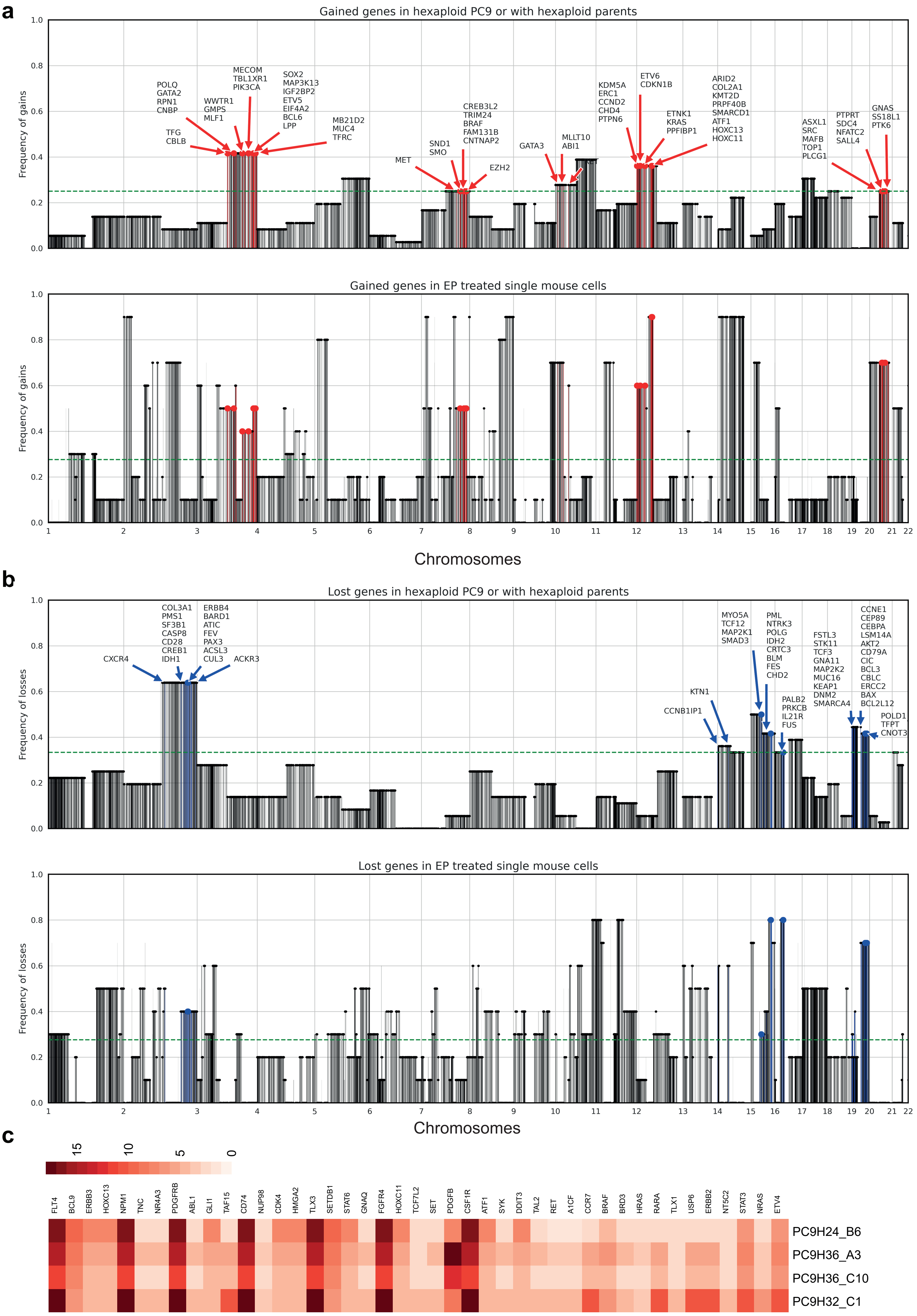
a
Gained genes in hexaploid PC9 or with hexaploid parents
Frequency of gains
POLQ
GATA2
RPN1
CNBP
TFG
CBLB
WWTR1
GMPS
MLF1
MECOM
TBL1XR1
PIK3CA
SOX2
MAP3K13
IGF2BP2
ETV5
EIF4A2
BCL6
LPP
MB21D2
MUC4
TFRC
MET
SND1
SMO
CREB3L2
TRIM24
BRAF
FAM131B
CNTNAP2
EZH2
GATA3
MLLT10
ABI1
KDM5A
ERC1
CCND2
CHD4
PTPN6
ETV6
CDKN1B
ETNK1
KRAS
PPFIBP1
ARID2
COL2A1
KMT2D
PRPF40B
SMARCD1
ATF1
HOXC13
HOXC11
ASXL1
SRC
MAFB
TOP1
PLCG1
PTPRT
SDC4
NFATC2
SALL4
GNAS
SS18L1
PTK6
Gained genes in EP treated single mouse cells
Frequency of gains
Chromosomes
b
Lost genes in hexaploid PC9 or with hexaploid parents
Frequency of losses
CXCR4
COL3A1
PMS1
SF3B1
CASP8
CD28
CREB1
IDH1
ERBB4
BARD1
ATIC
FEV
PAX3
ACSL3
CUL3
ACKR3
CCNB1IP1
KTN1
MYO5A
TCF12
MAP2K1
SMAD3
PML
NTRK3
POLG
IDH2
CRTC3
BLM
FES
CHD2
PALB2
PRKCB
IL21R
FUS
FSTL3
STK11
TCF3
GNA11
MAP2K2
MUC16
KEAP1
DNM2
SMARCA4
CCNE1
CEP89
CEBPA
LSM14A
AKT2
CD79A
CIC
BCL3
CBLC
ERCC2
BAX
BCL2L12
POLD1
TFPT
CNOT3
Lost genes in EP treated single mouse cells
Frequency of losses
Chromosomes
c
15
10
5
0
FLT4
BCL9
ERBB3
HOXC13
NPM1
TNC
NR4A3
PDGFRB
ABL1
GLI1
TAF15
CD74
NUP98
CDK4
HMGA2
TLX3
SETDB1
STAT6
GNAQ
FGFR4
HOXC11
TCF7L2
SET
PDGFB
CSF1R
ATF1
SYK
DDIT3
TAL2
RET
A1CF
CCR7
BRAF
BRD3
HRAS
RARA
TLX1
USP6
ERBB2
NT5C2
STAT3
NRAS
ETV4
PC9H24_B6
PC9H36_A3
PC9H36_C10
PC9H32_C1

Supplementary Data Fig. 10: Synteny of copy number changes in murine and cell models of EGFR tyrosine kinase inhibition resistance with Trp53/TP53 disruption. Synteny comparison analysis of copy-number gains and losses between resistant PC9 hexaploid subclones with hexaploid progenitors (n=36) and and EP resistant mouse tumors (n=10). **a**, The frequency of copy-number gains (y-axis) for every gene across the human chromosomes (x-axis) is displayed across the hexaploid PC9 resistant clones (top plot) and across the EP resistant mouse tumors (bottom plots), in which mouse genes have been mapped to the corresponding human gene through a synteny analysis. A threshold for defining recurrent gains in each of the two cohorts (dashed green line) has been computed based on a sampling and simulation method (Online Methods). Cancer genes that are recurrently gained in both hexaploid PC9 resistant subclones and EP resistant mouse tumors are highlighted (red). **b**, The frequency of copy-number losses (y-axis) for every gene across the human chromosomes (x-axis) is displayed across the hexaploid PC9 resistant clones (top plot) and across the EP resistant mouse tumors (bottom plots), in which mouse genes have been mapped to the corresponding human gene through a synteny analysis. A threshold for defining recurrent losses in each of the two cohorts (dashed green line) has been computed based on a sampling and simulation method (Online Methods). Cancer genes that are recurrently lost in both hexaploid PC9 resistant subclones and EP resistant mouse tumors are highlighted (blue). **c**, Absolute copy-numbers were estimated for four hexaploid resistant subclones. Source data are provided as a Source Data file.

Supplementary References.

1 Reiter, J. G. et al. An analysis of genetic heterogeneity in untreated cancers. Nat Rev Cancer 19, 639-650, doi:10.1038/s41568-019-0185-x (2019).
2 Dentro, S. C. et al. Characterizing genetic intra-tumor heterogeneity across 2,658 human cancer genomes. Cell 184, 2239-2254 e2239, doi:10.1016/j.cell.2021.03.009 (2021).

NSCLC - erlotinib					
	Response category New Lesion and Non-target L				
	No NL and no NTPD (N=136)	NL and no NTPD (N=43)	no NL and NTPD (N=19)	NL and NTPD (N=39)	Total (N=237)
	N (%)	N (%)	N (%)	N (%)	N (%)
**Response category Target Lesions**					
**Only responses**	25 (18.4)	0 (0.0)	0 (0.0)	0 (0.0)	25 (10.5)
**At least one response and other stable (no PD)**	23 (16.9)	3 (7.0)	0 (0.0)	1 (2.6)	27 (11.4)
**At least one response and at least one PD**	11 (8.1)	3 (7.0)	3 (15.8)	4 (10.3)	21 (8.9)
**Only stable lesions**	32 (23.5)	15 (34.9)	7 (36.8)	8 (20.5)	62 (26.2)
**Only stable and PD**	36 (26.5)	15 (34.9)	4 (21.1)	14 (35.9)	69 (29.1)
**All lesions PD**	9 (6.6)	7 (16.3)	5 (26.3)	12 (30.8)	33 (13.9)

NL = new lesion, NTPD = non-target progressive disease
Note: no NTPD includes patients without non-target lesions, patients achieving a non-target complete response and patients with stable non-target lesions

Supplementary Table 1
Summary of response data for erlotinib treated EORTC patients who had at least two lesions.

NSCLC - chemotherapy					
	Response category New Lesion and Non-target L				
	No NL and no NTPD (N=873)	NL and no NTPD (N=132)	no NL and NTPD (N=46)	NL and NTPD (N=41)	Total (N=1092)
	N (%)	N (%)	N (%)	N (%)	N (%)
**Response category Target Lesions**					
**Only responses**	201 (23.0)	10 (7.6)	2 (4.3)	0 (0.0)	213 (19.5)
**At least one response and other stable (no PD)**	333 (38.1)	29 (22.0)	10 (21.7)	8 (19.5)	380 (34.8)
**At least one response and at least one PD**	77 (8.8)	14 (10.6)	5 (10.9)	10 (24.4)	106 (9.7)
**Only stable lesions**	167 (19.1)	35 (26.5)	15 (32.6)	6 (14.6)	223 (20.4)
**Only stable and PD**	82 (9.4)	37 (28.0)	9 (19.6)	12 (29.3)	140 (12.8)
**All lesions PD**	13 (1.5)	7 (5.3)	5 (10.9)	5 (12.2)	30 (2.7)

NL = new lesion, NTPD = non-target progressive disease
Note: no NTPD includes patients without non-target lesions, patients achieving a non-target complete response and patients with stable non-target lesions.

Supplementary Table 2
Summary of response data for chemotherapy treated EORTC patients who had at least two lesions.

Cohort	Phase	Treatment	Trial Recruitment (n)	EGFR Mutation Status	Line of Therapy	Endpoint	PMID
AURA2	II	Osimertinib	199	T790M Positive	Second Line (following progression on prior EGFR TKI)	ORR	27751847
AURA3	III	Osimertinib	419	T790M Positive	Second Line (following progression on prior EGFR TKI)	PFS	27959700
AURA Extension	Phase II expansion of AURA phase I/II trial	Osimertinib	201	T790M Positive	Second Line (following progression on prior EGFR TKI)	ORR	28221867

Supplementary Table 3
Details of the patients and treatments involved in the AURA 2, AURA 3 and the phase II expansion of the AURA trial.
