# Supplementary material for: Mixed responses to targeted therapy driven by chromosomal instability through p53 dysfunction and genome doubling
Source: Nat Commun. 2024 Jun 13;15:4871. doi: 10.1038/s41467-024-47606-9 (PMC11176322; doi:10.1038/s41467-024-47606-9)
Supplement: Supplementary file 3 — Description of Additional Supplementary Files [file 41467_2024_47606_MOESM3_ESM.pdf]

Description of Additional Supplementary Files

Supplementary Data 1

Description: Summary of oncogenes and tumor suppressor genes identified as significantly recurrently affected by copy number gains and losses respectively, in both E and EP tumors in both mouse and human tumors.

Supplementary Data 2

Description: Summary of therapy regimens used for mouse erlotinib or chemotherapy treatment. Mutation calling and VAF

Supplementary Data 3

Description: List of genes identified as significantly gained or lost in resistant PC9 clones

Supplementary Data 4

Description: List of siRNA used.
