# Supplementary material for: Mixed responses to targeted therapy driven by chromosomal instability through p53 dysfunction and genome doubling
Source: Nat Commun. 2024 Jun 13;15:4871. doi: 10.1038/s41467-024-47606-9 (PMC11176322; doi:10.1038/s41467-024-47606-9)
Supplement: Supplementary file 8 — Reporting Summary [file 41467_2024_47606_MOESM8_ESM.pdf]

nature portfolio

Corresponding author(s): Eva Grönroos and Charles Swanton

Last updated by author(s): Feb 21, 2024

Reporting Summary

Nature Portfolio wishes to improve the reproducibility of the work that we publish. This form provides structure for consistency and transparency in reporting. For further information on Nature Portfolio policies, see our Editorial Policies and the Editorial Policy Checklist.

Statistics

For all statistical analyses, confirm that the following items are present in the figure legend, table legend, main text, or Methods section.

n/a	Confirmed	
☐	☒	The exact sample size (*n*) for each experimental group/condition, given as a discrete number and unit of measurement
☐	☒	A statement on whether measurements were taken from distinct samples or whether the same sample was measured repeatedly
☐	☒	The statistical test(s) used AND whether they are one- or two-sided<br>*Only common tests should be described solely by name; describe more complex techniques in the Methods section.*
☐	☒	A description of all covariates tested
☐	☒	A description of any assumptions or corrections, such as tests of normality and adjustment for multiple comparisons
☐	☒	A full description of the statistical parameters including central tendency (e.g. means) or other basic estimates (e.g. regression coefficient) AND variation (e.g. standard deviation) or associated estimates of uncertainty (e.g. confidence intervals)
☐	☒	For null hypothesis testing, the test statistic (e.g. *F*, *t*, *r*) with confidence intervals, effect sizes, degrees of freedom and *P* value noted<br>*Give P values as exact values whenever suitable.*
☒	☐	For Bayesian analysis, information on the choice of priors and Markov chain Monte Carlo settings
☒	☐	For hierarchical and complex designs, identification of the appropriate level for tests and full reporting of outcomes
☐	☒	Estimates of effect sizes (e.g. Cohen's *d*, Pearson's *r*), indicating how they were calculated

Our web collection on statistics for biologists contains articles on many of the points above.

Software and code

Policy information about availability of computer code

Data collection	No software was used to collect data
Data analysis	Prism software (version 10.1.2, Graph Pad Software Inc.)<br>R (version 3.6.2)<br>bwa mem (bwa v0.7.15)<br>Picard (v1.107, v2.18.11, v2.1.1)<br>FASTQC (v0.10.1, v0.11.5)<br>GATK (v4.1.3, 3.6, 2.8)<br>Scalpel(v0.5.3)<br>SAMtools (v1.3.1)<br>MuTect(v1.1.7)<br>MuTect2 (GATK v4.1.3)<br>Annovar (v58)<br>BedTools (v2.26)<br>platypus (v0.8.1)<br>DNAcopy (v1.54)<br>mclust algorithm, v5.4.5<br>Bowtie2 (v2.2.4)<br>BamUtil (v1.0.3)

AneuFinder (v1.8.0)
Varscan2 (v2.4.1)
BedTools (v2.26)
deepSNV (v1.18.1)

hhttps://github.com/zaccaria-lab/TP53loss_WGD

For manuscripts utilizing custom algorithms or software that are central to the research but not yet described in published literature, software must be made available to editors and reviewers. We strongly encourage code deposition in a community repository (e.g. GitHub). See the Nature Portfolio guidelines for submitting code & software for further information.

Data

Policy information about availability of data

All manuscripts must include a data availability statement. This statement should provide the following information, where applicable:

- Accession codes, unique identifiers, or web links for publicly available datasets
- A description of any restrictions on data availability
- For clinical datasets or third party data, please ensure that the statement adheres to our policy

Source data are provided with this manuscript. The sequencing data generated in this study have been deposited in ENA (https://www.ebi.ac.uk/ena/browser/home) with the accession numbers PRJEB55482, PRJEB55481 and PRJEB55479. Processed data (including copy number profiles and related analysis) for the E/EP mouse tumors and for the PC9 resistance cell lines are available in Zenodo at https://doi.org/10.5281/zenodo.10156620. The WES data (from the TRACERx study) used during this study has been deposited at the European Genome-phenome Archive, which is hosted by the European Bioinformatics Institute and the Centre for Genomic Regulation https://ega-archive.org/datasets/EGAS00001006494. Data (from the TRACERx study) used or analysed during this study are available through the Cancer Research UK and University College London Cancer Trials Centre (ctc.tracerx@ucl.ac.uk) for academic non-commercial research purposes only, and subject to review of a project proposal that will be evaluated by a TRACERx data access committee, entering into an appropriate data access agreement and subject to any applicable ethical approvals. Data from the TCGA and OncoSG can be found at https://www.cbioportal.org/ and https://src.gisapps.org/OncoSG/ respectively. Data from the AURA trials is available on request from AstraZeneca https://vivli.org/ourmember/astrazeneca/. Data from the San Francisco Clinical Cohort is available on request from Dr. Bivona. Biological materials are available on request

Human research participants

Policy information about studies involving human research participants and Sex and Gender in Research.

Reporting on sex and gender

No a priori analyses were specified based on sex. We did not have access to information regarding sex or gender for the patients enrolled in the AstraZeneca trials or the EORTC RECIST database. Additional data used in this manuscript is taken from other published cohorts which have reported on sex distribution. We have not performed any post hoc subgroup analyses based on sex. The conclusions of this manuscript should considered applicable to patients with EGFR mutation positive lung cancer irrespective of sex.

The OncoSG cohort is predominantly composed of patients recruited in Asia in contrast to the TRACERx and TCGA cohorts which recruited patients with predominantly European ancestry. The AURA trials were global studies recruiting from multiple ethnicities. We do not have access to information regarding ethnicity for individual patients except for those in the TRACERx and TCGA cohorts which have been reported elsewhere.

Population characteristics

Please note that the study started recruiting patients in 2016, when TNM version 7 was standard of care. The up-to-date inclusion/exclusion criteria now utilizes TNM version 8.

TRACERx inclusion and exclusion criteria:

Inclusion Criteria:
Written Informed consent
Patients ≥18 years of age, with early stage I-IIIB disease (according to TNM 8th edition) who are eligible for primary surgery.
Histopathologically confirmed NSCLC, or a strong suspicion of cancer on lung imaging necessitating surgery (e.g. diagnosis determined from frozen section in theatre)
Primary surgery in keeping with NICE guidelines planned
Agreement to be followed up at a TRACERx site
Performance status 0 or 1
Minimum tumor diameter at least 15mm to allow for sampling of at least two tumour regions (if 15mm, a high likelihood of nodal involvement on pre-operative imaging required to meet eligibility according to stage, i.e. T1N1-3)

Exclusion Criteria:
Any other* malignancy diagnosed or relapsed at any time, which is currently being treated (including by hormonal therapy).
Any other* current malignancy or malignancy diagnosed or relapsed within the past 3 years**.
*Exceptions are: non-melanomatous skin cancer, stage 0 melanoma in situ, and in situ cervical cancer
**An exception will be made for malignancies diagnosed or relapsed more than 2, but less than 3, years ago only if a pre-operative biopsy of the lung lesion has confirmed a diagnosis of NSCLC.

Psychological condition that would preclude informed consent
Treatment with neo-adjuvant therapy for current lung malignancy deemed necessary
Post-surgery stage IV
Known Human Immunodeficiency Virus (HIV), Hepatitis B Virus (HBV), Hepatitis C Virus (HCV) or syphilis infection.
Sufficient tissue, i.e. a minimum of two tumor regions, is unlikely to be obtained for the study based on pre-operative imaging

Patient ineligibility following registration
There is insufficient tissue
The patient is unable to comply with protocol requirements
There is a change in histology from NSCLC following surgery, or NSCLC is not confirmed during or after surgery.
_Change in staging to IIIC or IV following surgery
_The operative criteria are not met (e.g. incomplete resection with macroscopic residual tumors (R2)). Patients with microscopic residual tumors (R1) are eligible and should remain in the study
_Adjuvant therapy other than platinum-based chemotherapy and/or radiotherapy is administered.

Recruitment

TRACERx: Patients seen with a new diagnosis of lung cancer in lung cancer units across the United Kingdom, according to the eligibility criteria above, were recruited. No selection bias has been identified to date.

AURA trial recruitment was multi-national as reported in the clinical trial summary of these studies.

Ethics oversight

The TRACERx study was approved by the NRES Committee London with the following details:
Study title: TRAcking non small cell lung Cancer Evolution through therapy (Rx)
REC reference: 13/LO/1546
Protocol number: UCL/12/0279
IRAS project ID: 138871

Clinical data was anonymised and all observational or interventional trials had the relevant ethical approval as indicated in the primary reports.

Note that full information on the approval of the study protocol must also be provided in the manuscript.

Field-specific reporting

Please select the one below that is the best fit for your research. If you are not sure, read the appropriate sections before making your selection.

☒ Life sciences ☐ Behavioural & social sciences ☐ Ecological, evolutionary & environmental sciences

For a reference copy of the document with all sections, see nature.com/documents/nr-reporting-summary-flat.pdf

Life sciences study design

All studies must disclose on these points even when the disclosure is negative.

Sample size

EGFR-mutant positive patients identified within the total cohort of 421 patients that represents the half-way point of the TRACERx longitudinal study. In total 25 of 421 patients were found to harbor clonal EGFR mutations and were analysed in this study.
PFS of 117 patients from the AURA2 and AURA3 clinical trials, as well as the AURA trial phase II expansion cohort were analyzed.
Response dynamics were examined in 68 patients from the AURA cohorts. The sample size of the TRACERx cohort was 421 patients of which 25 had an EGFR mutation. For the UCSF cohort was 84 patients of which 54 had a TP53 co-mutation.

Sample size for in vivo experiments was chosen based on pilot experiments and the effect size observed in the survival analysis. No formal power calculations were done for the tumour size analysis, as the numbers were based on the survival data. Exact numbers of animals for each experiment are in the provided in the source data file.

Data exclusions

PFS analysis of the combined AURA cohorts was restricted to patients with available tissue-based tumor somatic analysis (n=117). Response dynamics were examined in patients who had at least two metastatic lesions at the baseline scan, had tissue-based tumor somatic analysis and had consented to share longitudinal follow-up imaging.

For in vivo experiments some animals were excluded from the survival analysis as they had to be culled prematurely due to unrelated reasons (i.e fight wounds, over-grooming). One animal was excluded as it developed a lymphoma. Two mice were excluded from some of the single cell analyses due to low sequencing coverage.

Replication

For in vitro studies three independent replicates were performed for all experiments and were successful. For in vivo experiment animal numbers are indicated in the text and source data file.

Randomization

Patients in the AURA trial were randomised as per the trial protocol. For the in vivo analyses mice were randomly allocated to treated or untreated groups based on genotype.

Blinding	The TP53 mutation status of the patients was unknown to the scientist performing lesion measurements in the patient data anlyses. For the in vivo studies the time for culling animals (survival analysis) was called by animal technicians which were blinded to animal genotypes. The analysis of the in vivo imaging data was blinded for the genotype of the animals.

Reporting for specific materials, systems and methods

We require information from authors about some types of materials, experimental systems and methods used in many studies. Here, indicate whether each material, system or method listed is relevant to your study. If you are not sure if a list item applies to your research, read the appropriate section before selecting a response.

Materials & experimental systems

n/a	Involved in the study	
☐	☒	Antibodies
☐	☒	Eukaryotic cell lines
☒	☐	Palaeontology and archaeology
☐	☒	Animals and other organisms
☐	☒	Clinical data
☒	☐	Dual use research of concern

Methods

n/a	Involved in the study	
☒	☐	ChIP-seq
☒	☐	Flow cytometry
☒	☐	MRI-based neuroimaging

Antibodies

Antibodies used	Antibody Company Catalogue No. Clone<br>EGFR L858R CST 3197S 43B2<br>anti Ki67 Abcam ab15580 N/A
Validation	The antibodies used have been validated accordingly to manufacturer's instructions. Positive control sections were used to validate all antibodies for IHC staining.

Eukaryotic cell lines

Policy information about cell lines and Sex and Gender in Research

Cell line source(s)	PC9 cells were obtained from Cell Services at The Francis Crick Institute, UK
Authentication	Cell identity is confirmed by short tandem repeat profiling performed by Cell Services at The Francis Crick Institute, UK
Mycoplasma contamination	All cells received from Cell Services at The Francis Crick Institute, UK have been screened for mycoplasma contamination and tested negative before use.
Commonly misidentified lines<br>(See ICLAC register)	No commonly misidentified cell lines were used in this study

Animals and other research organisms

Policy information about studies involving animals; ARRIVE guidelines recommended for reporting animal research, and Sex and Gender in Research

Laboratory animals	EGFR-L858R [Tg(tet-O-EGFR*L858R)56Hev], R26tTA [Gt(ROSA)26Sortm1(tTA)Roos] and Trp53fl/fl [Trp53tm1Brn] were crossed to generate experimental animals. Rosa26tTaLSL/tet(O)EGFRL858R and Rosa26tTaLSL/tet(O)EGFRL858R/Trp53flox/flox mice were back crossed onto a C57Bl6/J background. We used both male and female mice between 2.5-6.5 months of age. All mice were bred in house at the Francis Crick Biological Research Facility, according to UK Home Office Regulations. Light cycles were 7am-7pm, the ambient temperature and humidity was maintained at 21 degrees and 50%, respectively.
Wild animals	No wild animals were used in this study
Reporting on sex	Care was taken to have sex balanced animal cohorts.
Field-collected samples	N/A
Ethics oversight	All animal regulated procedures were approved by The Francis Crick Institute BRF Strategic Oversight Committee, incorporating the Animal Welfare and Ethical Review Body, conforming with UK Home Office guidelines and regulations under the Animals (Scientific Procedures) Act 1986 including Amendment Regulations 2012.

Note that full information on the approval of the study protocol must also be provided in the manuscript.

Clinical data

Policy information about clinical studies

All manuscripts should comply with the ICMJE guidelines for publication of clinical research and a completed CONSORT checklist must be included with all submissions.

Clinical trial registration	TRACERx Lung https://clinicaltrials.gov/ct2/show/NCT01888601, approved by an independent Research Ethics Committee, 13/LO/1546<br>AURA2 and AURA3 clinical trials, as well as the AURA trial phase II expansion cohort, identifiers: NCT02094261, NCT02151981 and NCT01802632.
Study protocol	https://clinicaltrials.gov/ct2/show/NCT01888601
Data collection	TRACERx : Clinical and pathological data is collected from patients during study follow up - this period is a minimum of five years. Data collection is overseen by the sponsor of the study (Cancer Research UK & UCL Cancer Trials Centre) and takes place in hospitals across the United Kingdom. A centralised database called MACRO is used for this purpose.
Outcomes	TRACERx: Disease-free outcome data were gathered for all patients. Last updated in 15 June 2021
